# Metabolomics Evaluation of Serum Markers for Cachexia and Their Intra-Day Variation in Patients with Advanced Pancreatic Cancer

**DOI:** 10.1371/journal.pone.0113259

**Published:** 2014-11-20

**Authors:** Yutaka Fujiwara, Takashi Kobayashi, Naoko Chayahara, Yoshinori Imamura, Masanori Toyoda, Naomi Kiyota, Toru Mukohara, Shin Nishiumi, Takeshi Azuma, Masaru Yoshida, Hironobu Minami

**Affiliations:** 1 Division of Medical Oncology/Hematology, Kobe University Graduate School of Medicine, Kobe, Japan; 2 Division of Gastroenterology, Kobe University Graduate School of Medicine, Kobe, Japan; 3 Cancer Center, Kobe University Hospital, Kobe, Japan; 4 Division of Metabolomics Research, Kobe University Graduate School of Medicine, Kobe, Japan; INIA, Spain

## Abstract

**Purpose:**

Cancer cachexia is a multifactorial syndrome characterized by progressive loss of weight and muscle atrophy. Using metabolomics, we investigated serum markers and their intra-day variation in advanced pancreatic cancer patients with cachexia.

**Methods:**

Patients were enrolled in two groups: those with or without cachexia. Blood samples collected at 6:30 AM, 11:30 AM, 4:30 PM, and 9:30 PM were analyzed using metabolomics, and serum levels of IL-6, TNF-α, and leptin were measured and compared between the two groups. Intra-day variation was then evaluated.

**Results:**

Twenty-one patients were enrolled in total. In the cachexia group (n = 9), median body weight loss rate over 6 months was greater, performance status was poorer, and anorexia was more severe than in the non-cachexia group (n = 12). Each metabolites level showed substantial intra-day variation, and some of them displayed significant differences between the two groups. Levels of paraxanthine remained markedly lower in the cohort with cachexia at all measurement points. Besides, median IL-6 and TNF-α levels appeared higher and leptin concentration appeared lower in the cachexia group, albeit without statistical significance.

**Conclusion:**

Some metabolites and some serological marker levels were affected by cancer cachexia. Although paraxanthine levels were consistently lower in patients with cachexia, we identified that many metabolites indicated large intra- and inter-day variation and that it might be necessary to pay attention to intra-day variation in metabolomics research.

## Introduction

Cachexia is a multifactorial syndrome characterized by progressive loss of weight and muscle atrophy that cannot be fully reversed by conventional nutritional support, thereby leading to progressive functional impairment [1]. Its pathophysiology is considered to involve a negative protein and energy balance driven by a variable combination of reduced food intake and abnormal metabolism. The agreed diagnostic criterion for cachexia is weight loss of greater than 5%, or weight loss greater than 2% in individuals already showing depletion, according to current body weight and height (body mass index <20 kg/m^2^) or skeletal muscle mass [2].

Cachexia is reported in approximately 80% of advanced pancreatic cancer patients who typically develop a decreased dietary intake and a range of symptoms such as anorexia, early satiety, anxiety, and depression [3]. Cachexia has been shown to worsen prognosis and has also been associated with impairment of physical function, increased psychological distress, a reduction in tolerance of and response to therapy, a decrease in quality of life, and reduced duration of survival [4], [5]. However, while substantial research is currently focused on determining the mechanism behind cachexia development, no precise understanding has yet been obtained.

Fasting hormones, such as leptin and ghrelin; pro-inflammatory cytokines, such as tumor necrosis factor-alpha (TNF-α), interferon gamma, and interleukin 6 (IL-6); insulin-like growth factor-1 (IGF-1); and the tumor-secreted proteolysis-inducing factor have all been implicated to some extent in cachexia development [6]–[9]. An improved understanding of the integrative physiology of cancer cachexia may thus yield further novel therapeutic approaches.

Metabolomics (metabolome analysis) may prove useful in identifying the dynamic metabolic response of a living system to pathophysiological stimuli [10], [11]. In metabolite profiling, the selected metabolites in a particular environment are identified and then subjected to quantitative or semi-quantitative assessment. This approach is useful for facilitating understanding of known metabolic pathways and biological alterations in mammalian homeostasis and living systems in pathophysiology [12].

Metabolism is known to be subject to circadian rhythms. The circadian timekeeping system in mammalian homeostasis is a hierarchical multi-oscillator network, with the suprachiasmatic nucleus acting as the central pacemaker [13], [14], synchronizing to daily light-dark cycles and coordinating circadian metabolism and physiology. A comprehensive understanding of the pathophysiology of cancer cachexia may require consideration of the influence of intra-day variation [15].

Here, we investigated the difference in serum metabolite levels in pancreatic cancer patients with and without cachexia and analyzed the pattern and intra-day variation in metabolite levels using metabolomics.

## Methods

### Patient selection criteria

The study (trial registration ID: UMIN000002384) was conducted in hospitalized patients at Kobe University Hospital with pancreatic cancer that was locally advanced or metastatic and not amenable to curative surgical resection. Additional eligibility criteria were age ≥20 years, histologically confirmed adenocarcinoma or adenosquamous carcinoma of the pancreas, and adequate organ function (serum total bilirubin <1.5× upper limit of normal [ULN], aspartate aminotransferase [AST] <2.5×ULN, alanine aminotransferase [ALT] <2.5×ULN, and serum creatinine <1.5×ULN). No chemotherapy in the previous 7 days, no surgery or definitive irradiation in the previous 4 weeks, and palliative irradiation in the previous 2 weeks were permitted. Other exclusion criteria included active multiple primary cancer, serious pre-existing medical condition such as uncontrolled infection, diabetes mellitus, and concomitant use of steroids.

To discern small variations in metabolites, this study included cohorts with and without cachexia, defined as follows: the cohort with cachexia included those with an Eastern Cooperative Oncology Group performance status (ECOG PS) 1 to 4, grade 1 to 4 anorexia, and weight loss greater than 10% over the past 6 months; while that without cachexia included patients with a ECOG PS 0 to 2, grade 0 to 1 anorexia, serum albumin levels exceeding 3.5 mg/dL, and weight loss less than 5% over the past 6 months. Patients who didn't meet these cohort criteria were also excluded in this study.

All patients provided written informed consent, and study approval was obtained from the Institutional Review Board of Kobe University Hospital.

### Objectives and outcomes

The objective of this observational study was to investigate the difference in serum metabolite levels between pancreatic cancer patients with and without cachexia and to explore the pattern and intra-day variations in metabolite levels using metabolomics. Eligible subjects were assigned to either cohort with cachexia or that without cachexia. Primary endpoint was identification of cachexia-related metabolites using metabolomics. Secondary endpoints were intra-day variation in the metabolites involved in cachexia and changes in the level of serological markers involved in cachexia, such as inflammatory cytokines (e.g. IL-6, TNFα) and leptin, in the presence or absence of cachexia. Patient characteristics and medication information were recorded throughout the study. Adverse events were evaluated using the CTCAE v4.0.

The present study featured 10 subjects per group, as although this study was exploratory in nature and therefore involved no statistical rationale for sample size calculation, preceding studies on metabolomics have indicated significant results with a sample size of approximately 10 subjects per group. However, if a more useful analysis method found is found that may be implemented in the study, the analysis methods may be altered and the necessary sample size recalculated.

### Serum collection and preparation

Blood samples from hospitalized subjects were collected at 6:30 AM (after waking in early morning), 11:30 AM (late morning), 4:30 PM (early evening), and 9:30 PM (before retiring at night) at equal intervals in a single day to analyze intra-day variation. After collection of whole blood, samples were allowed to clot at room temperature, and serum was separated by centrifugation at 3,000×g for 10 min at 4°C and stored at −80°C until use.

To extract low-molecular-weight metabolites, 50 µl of serum was mixed with 250 µl of a solvent mixture (MeOH:H_2_O:CHCl_3_ = 2.5∶1∶1) containing 10 µl of 0.5 mg/ml 2-isopropylmalic acid (Sigma-Aldrich, Tokyo, Japan) dissolved in distilled water, and then the solution was shaken at 1,200 rpm for 30 min at 37°C before being centrifuged at 16,000×g for 5 min at 4°C. A total of 225 µl of the obtained supernatant was transferred to a clean tube, and 200 µl of distilled water was added. After mixing, the solution was centrifuged at 16,000×g for 5 min at 4°C, and 250 µl of the resultant supernatant was transferred to a clean tube before being lyophilized using a freeze dryer. For oximation, 40 µl of 20 mg/ml methoxyamine hydrochloride (Sigma-Aldrich) dissolved in pyridine was mixed with a lyophilized sample, and the mixture was shaken at 1,200 rpm for 90 min at 30°C. N-methyl-N-trimethylsilyltrifluoroacetamide (MSTFA; 20 µl) (GL Science, Tokyo, Japan) was then added for derivatization, and the mixture was incubated at 1,200 rpm for 30 min at 37°C. The mixture was then centrifuged at 16,000×g for 5 min at 20°C, and the resultant supernatant was subjected to gas chromatography-mass spectrometry (GC/MS) measurement.

Serum leptin levels were measured via radioimmunoassay as described previously in SRL Inc. (Tokyo, Japan) [16], [17]. The limit of sensitivity was 0.5 ng/ml, and the intra- and interassay coefficients of variation both ranged from 2.5% to 5.0% over the sample concentration range. Serum IL-6 was detected using a Chemiluminescent Enzyme Immunoassay from SRL Inc. (Tokyo, Japan) [18]. The limit of sensitivity was 4.0 pg/ml, and the intra- and interassay coefficients of variation ranged from 2.2% to 3.8% and 3.6% to 8.6%, respectively, over the sample concentration range. Serum TNFα was detected using an Enzyme-Linked ImmunoSorbent Assay from SRL Inc. (Tokyo, Japan). Assay results ranged from 0.6 to 2.8 pg/ml.

### GC/MS analysis and data processing

GC/MS analysis was performed using a GCMS-QP2010 Ultra (Shimadzu Co., Kyoto, Japan) with a fused silica capillary column (CP-SIL 8 CB low bleed/MS; inner diameter, 30 mm×0.25 mm; film thickness, 0.25 µm; Agilent Co., Palo Alto, CA, USA), in accordance with a previously described method [19]. The front inlet temperature was 230°C, and the flow rate of helium gas through the column was 39.0 cm/sec. The column temperature was held at 80°C for 2 min and then raised by 15°C/min to 330°C and held there for 6 min. The transfer line and ion-source temperatures were 250°C and 200°C, respectively. Twenty scans per second were recorded over the mass range 85–500 m/z using the Advanced Scanning Speed Protocol (ASSP, Shimadzu Co.).

Data processing was performed in accordance with the methods described in previous reports [19], [20]. Briefly, the MS data were exported in netCDF format, and peak detection and alignment were performed using MetAlign software (Wageningen UR, The Netherlands). The resultant data were then exported in CSV format and analyzed using in-house analytical software. For semi-quantification, the peak height of each ion was calculated and normalized to the peak height of 2-isopropylmalic acid as an internal standard. Names were assigned to each metabolite peak based on the method described in a previous report [20].

### Statistical analysis

Data are expressed as median and range. Levels of serological markers and metabolites between the cohorts with and without cachexia were compared using the Mann-Whitney U test or Wilcoxon Signed-Rank test. Presence of intra-day variances in metabolite levels was then determined using Kruskal-Wallis multiple comparison (z-test) with bonferroni correction to compare values for each metabolite across all four time points assessed. Survival time was estimated using the Kaplan-Meier method. All statistical analyses were performed using NCSS 2007 software (NCSS, LLC. Kaysville, UT, USA).

## Results

### Patients' characteristics

**Figure 1:**
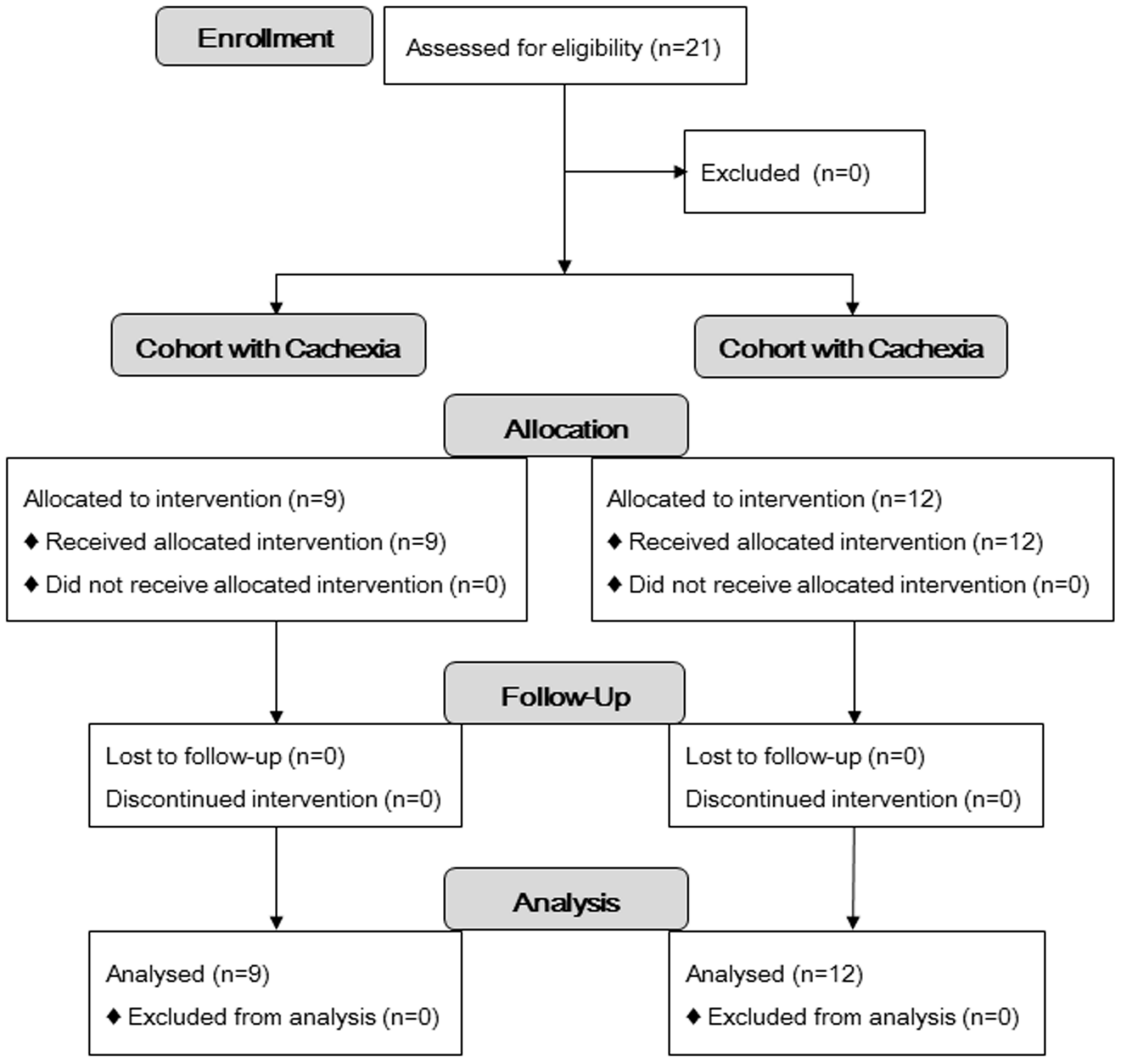
CONSORT Flow Diagram.

From December 2009 to July 2011, 21 patients were enrolled in this study: 9 with cachexia and 12 without (Figure 1). Clinical characteristics are summarized in Table 1. Median body weight loss rates over 6 months in the cohorts with and without cachexia were 13.4% and 2.5%, respectively (p = 0.0001). Of the 21 patients enrolled, 19 had no history of prior treatment. No significant differences were noted in levels of tumor markers, LDH, CRP, or HbA1c between the cohorts. However, levels of total cholesterol and LDL cholesterol were significantly lower in the cohort with cachexia. Eight patients in the cohort with cachexia and 8 in that without were male in the present study. Median ages in the cohorts with and without cachexia were 66.5 (range, 36 to 77) and 68.5 (39 to 76) years, respectively (p = 0.98). No severe adverse events or unintended effects were noted among any participants in the present study.

**Table 1:** Patient characteristics.

		With Cachexia	Without Cachexia	p-value
		n = 9	n = 12	
Age	(years)	72 (39–76)	64.5 (36–77)	0.57
Gender	Male/Female	8/1	8/4	0.25
PS	0/1/2/3	0/5/3/1	4/8/0/0	0.005
Stage	IVA/IVB	5/4	5/7	0.54
BW loss rate	(%)	13.4 (10.2–30.8)	2.5 (−5.2–4.6)	0.0001
Body weight	(kg)	55.6 (45.0–77.0)	57.5 (44.6–77.3)	0.48
Body mass index		22.6 (18.2–25.8)	19.7 (16.5–26.1)	0.15
Anorexia	Gr 0/1/2/3	0/8/0/1	5/7/0/0	0.02
Prior treatment	(No/Yes)	8/1	11/1	0.83
Laboratory data				
WBC	(/µL)	5100 (4100–17600)	5850 (3000–111600)	0.97
Hb	(g/dL)	11.9 (9.3–15.2)	13.7 (11.3–14.6)	0.20
TP	(g/dL)	6.4 (4.8–6.8)	6.7 (6.0–7.5)	0.15
Alb	(g/dL)	3.2 (2.1–4.7)	3.9 (3.5–4.4)	0.14
LDH	(U/L)	169 (125–228)	179 (127–340)	0.27
TChol	(mg/dL)	131 (99–167)	187 (136–205)	0.001
LDL	(mg/dL)	72 (45–91)	100 (71–143)	0.02
HDL	(mg/dL)	42 (22–73)	53 (29–93)	0.20
TG	(mg/dL)	90 (44–112)	112 (62–159)	0.09
CRP	(mg/dL)	1.9 (0.1–2.64)	0.55 (0.1–6.97)	0.89
HbA_1c_	(%)	5.7 (4.8–6.5)	6.4 (4.6–7.3)	0.11
Tumor marker				
CEA	(ng/mL)	5.0 (1.4–29.2)	4.7 (2.0–883.7)	1.00
CA19-9	(U/mL)	271 (5–1974)	379 (1–80673)	0.43
DUPAN-2	(U/mL)	560 (33–230000)	605 (25–140000)	0.89

Abbreviations: PS, Eastern Cooperative Oncology Group performance status; BW loss rate, body weight loss rate over 6 months; TChol, total Cholesterol; LDL, low-density lipoprotein; HDL, high-density lipoprotein; TG, triglyceride; CRP, C-reactive protein.

### Treatment and survival

After enrolling in this study, all 19 patients who had had no prior treatment subsequently received chemotherapy (n = 15) or chemoradiotherapy (n = 4). Regimens used in the 15 patients treated with chemotherapy were gemcitabine monotherapy (n = 13) and combination therapy of gemcitabine plus TS-1, an oral fluoropyrimidine prodrug (n = 2). In the four patients treated with chemoradiotherapy for locally advanced cancer, the regimens used (as part of another clinical study) were concurrent chemoradiotherapy with gemcitabine plus TS-1 (n = 2) and heavy-particle radiotherapy with gemcitabine (n = 2). The remaining 2 patients who had had prior treatment received best supportive care after enrolling in this study.

**Figure 2:**
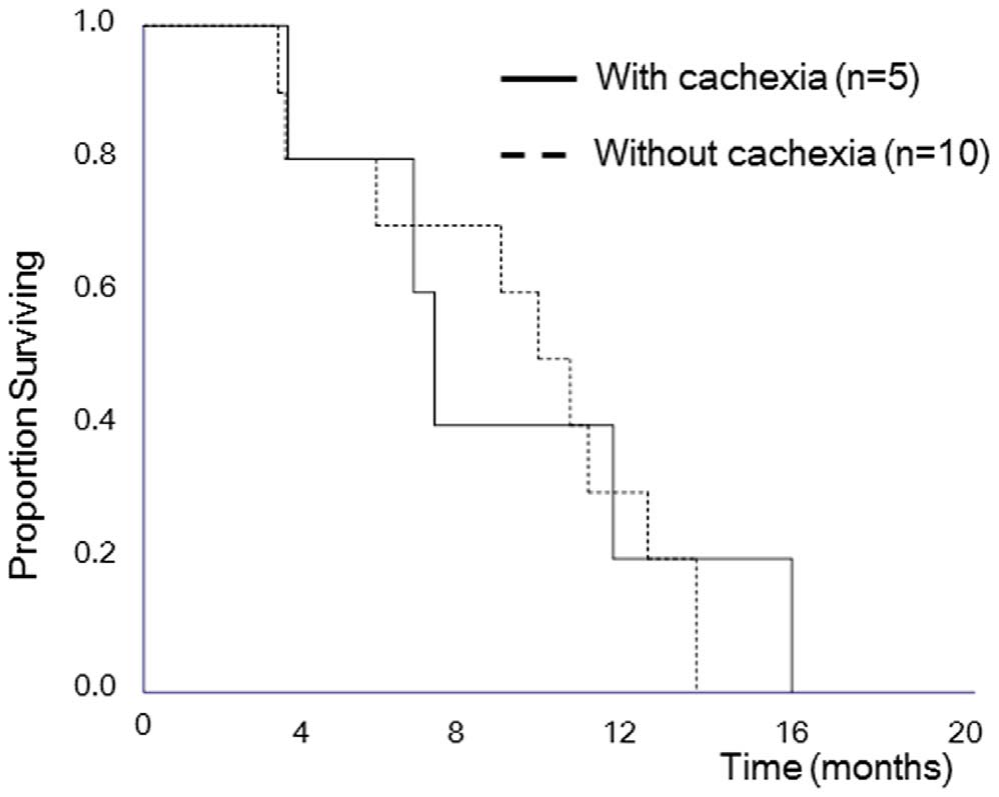
Kaplan-Meier curve for overall survival in patients who had no prior treatment.

We analyzed survival in the 19 patients who had received no prior treatment. The median survival time in patients with stage IVA (n = 9) and stage IVB (n = 10) was 15.6 and 7.0 months (logrank test, p<0.001), respectively, while that in patients treated with chemotherapy (n = 15) and chemoradiotherapy (n = 4) was 9.5 and 19.7 months (logrank test, p = 0.004) respectively. Although this study was too small to detect survival difference, the median survival time in patients who received chemotherapy was 7.0 months with cachexia (n = 5) and 9.5 months without (n = 10) (logrank test, p = 0.85), and 1-year survival rates were 20.0% and 30.0%, respectively (Figure 2).

### Metabolomics

In our GC/MS-based metabolomics analysis system, which mainly targeted water-soluble metabolites, 124 metabolites were detected in subjects' serum samples (Table S1). Of these 124 metabolites, 1 metabolite, namely 2-isopropylmalic acid, was used as an internal standard, and 8 were probably extracted from non-serum source, for example from eppendorf tubes. These nine metabolites were therefore excluded from subsequent analyses.

**Figure 3:**
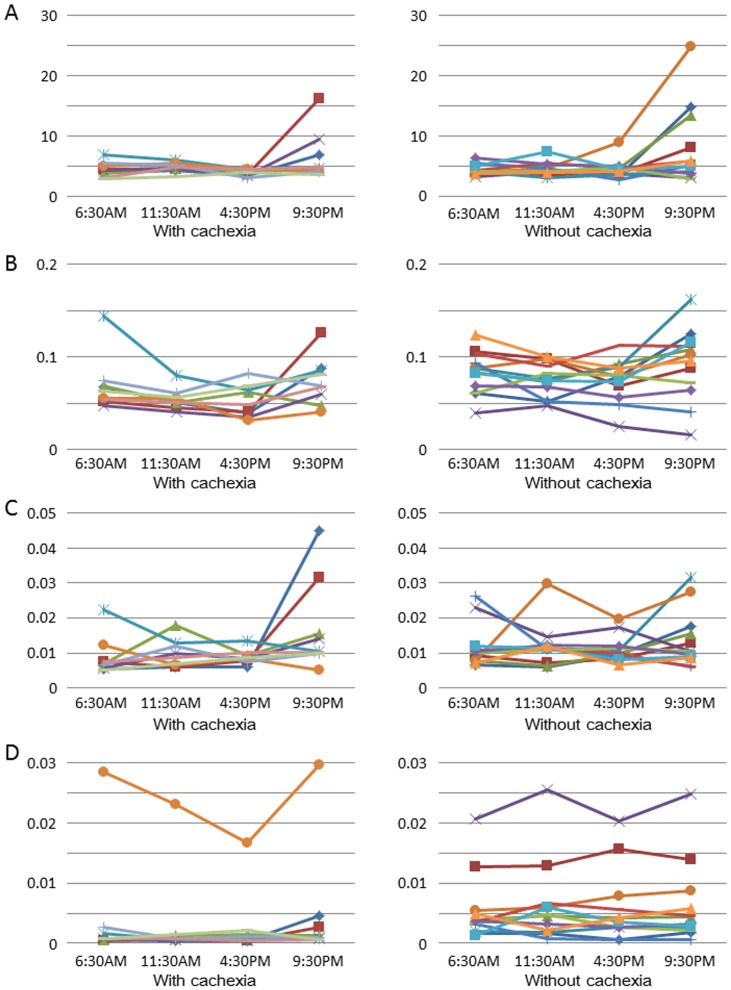
Intra-day variation in A) lactic acid, B) alanine, C) catechol, and D) paraxanthine.

**Figure 4:**
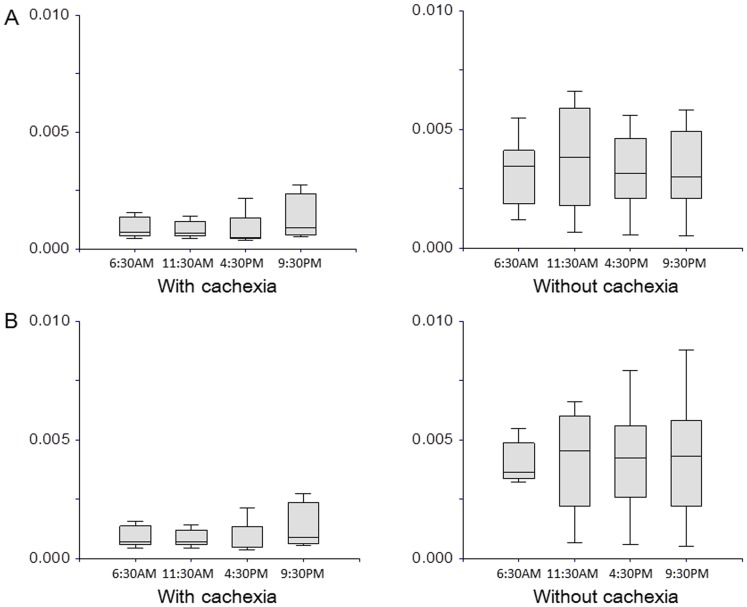
Intra-day variation in paraxanthine. A. In all patients. At 6:30 AM: 0.75×10^−3^ (95% CI, 0.28–2.7×10^−3^) in patients with cachexia vs. 3.61×10^−3^ (1.91–5.48×10^−3^) pg/ml in those without cachexia, p = 0.35; TNF-α: 7.1 (1.2 to 30.2) vs. 3.3 (1.2 to 30.0) pg/ml, p = 0.27; leptin: 2.4 (1.0 to 9.8) vs. 4.0 (2.0 to 9.9) ng/ml, p = 0.27, respectively. B. In male patients. IL-6: 15.2 (range, 2.6 to 23.4) in patients with cachexia vs. 9.4 (1.4 to 28.7) pg/ml in those without cachexia, p = 0.57; TNF-α: 12.4 (1.2 to 29.0) vs. 3.2 (1.4 to 28.6) pg/ml, p = 0.29; leptin: 2.3 (1.0 to 8.8) vs. 3.45 (2.0 to 2.8) ng/ml, p = 0.34, respectively.

Kruskal-Wallis multiple comparison (z-test) with bonferroni correction was used to evaluate changes in metabolite levels at four different time points throughout the day. These analyses showed that the levels of 60 of the 115 evaluated metabolites differed significantly between those means at any 1 of 4 time points. Univariate analysis of the 115 metabolites identified considerable inter-individual variability of levels of some metabolites throughout a single day. Figure 3 describes representative metabolites, including lactic acid, alanine, catechol, and paraxanthine. Metabolites with significant differences in intra-day levels on univariate analysis were (fold change in levels in the cohort with cachexia to that without, p-value): at 6:30 AM, catechol (0.70, p = 0.04) and paraxanthine (0.21, p = 0.006); at 11:30 AM, valine (0.68, p = 0.01), proline (0.66, p = 0.049), p-hydroxybenzoic acid (0.79, p = 0.03), and paraxanthine (0.16, p = 0.006); and at 4:30 PM, valine (0.62, p = 0.02) and paraxanthine (0.13, p = 0.007). Although differences in levels of paraxanthine were not statistically significant (0.26, p = 0.08) at 9:30 PM, the levels were clearly lower in the cohort with cachexia at all time points (Figure 4). Of note, the female patient with outlying paraxanthine values in the cohort with cachexia was the study's only heavy drinker of coffee, which is a metabolic substrate of paraxanthine (Figure 3 D).

### Serological markers

**Figure 5:**
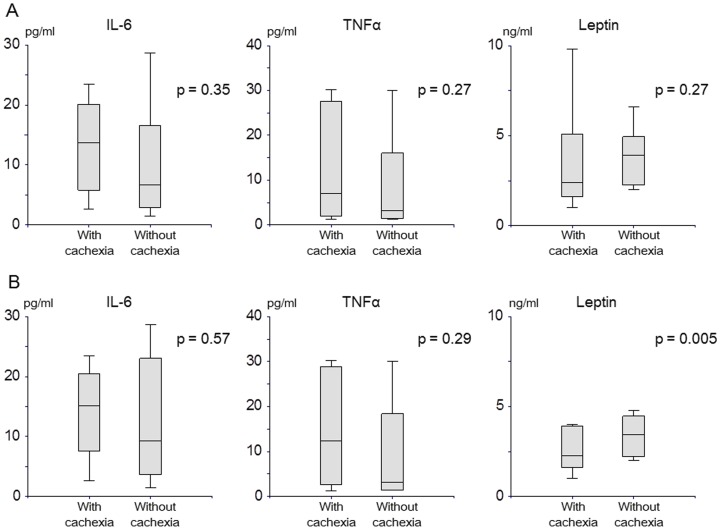
Serological markers between patients with cachexia and those without cachexia. A. In all patients. IL-6: 13.8 (range, 2.6 to 23.4) in patients with cachexia vs. 6.8 (1.4 to 28.7) pg/ml in those without cachexia, p = 0.35; TNF-α: 7.1 (1.2 to 30.2) vs. 3.3 (1.2 to 30.0) pg/ml, p = 0.27; leptin: 2.4 (1.0 to 9.8) vs. 4.0 (2.0 to 9.9) ng/ml, p = 0.27, respectively. B. In male patients. IL-6: 15.2 (range, 2.6 to 23.4) in patients with cachexia vs. 9.4 (1.4 to 28.7) pg/ml in those without cachexia, p = 0.57; TNF-α: 12.4 (1.2 to 29.0) vs. 3.2 (1.4 to 28.6) pg/ml, p = 0.29; leptin: 2.3 (1.0 to 8.8) vs. 3.45 (2.0 to 2.8) ng/ml, p = 0.34, respectively.

**Figure 6:**
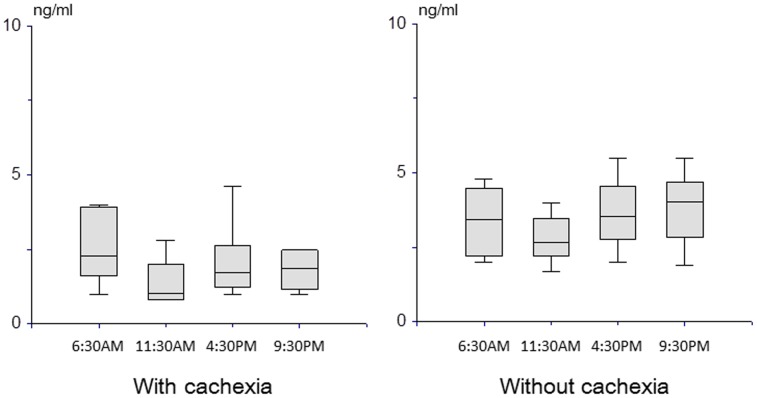
Intra-day variation in leptin.

Median levels of IL-6, TNF-α, and leptin at 6:30 AM didn't differ significantly between the cohorts with and without cachexia, and the observed trends in level fluctuations were similar to those previously reported (Figure 5A) [7] [9]. Because serum serological marker levels in healthy volunteers vary substantially depending on gender and age [21], we analyzed values in men. In the 16 male patients, median levels of IL-6, TNF-α, and leptin at 6:30 AM didn't differ significantly between the cohorts with and without cachexia (Figure 5B). Leptin concentrations during the day remained lower in the cohort with cachexia than in that without (Figure 6). However, median leptin levels did show substantial intra-day variation; 2.4 (range, 1.0 to 9.8) ng/ml and 1.1 (0.8 to 4.4) ng/ml at 6:30 AM and 11:30 respectively for patients with cachexia (p = 0.003), and 3.95 (2.0 to 9.9) ng/ml and 3.1 (1.5 to 7.8) ng/ml at 6:30 AM and 11:30 respectively for those without cachexia (p = 0.03).

## Discussion

Cancer cachexia is a multifactorial syndrome characterized by body weight loss and muscle and adipose tissue wasting and inflammation, and is often associated with anorexia. Abnormalities commonly associated with cachexia include alterations in carbohydrate, lipid and protein metabolism. A substantial amount of research is currently focused on determining the mechanism behind cachexia development, with several factors considered to be putative mediators of cancer anorexia, including hormones (e.g. leptin), neuropeptides, cytokines (e.g. IL-1, IL-6, and TNF-α), and neurotransmitters (e.g. serotonin and dopamine). Here, we examined levels of typical serological factors, such as IL-6, TNF-α, and leptin, and used metabolomics to identify metabolites associated with cachexia in patients with pancreatic cancer and investigate intra-day variations in levels of these metabolites. To our knowledge, this is the first study to analyze cancer cachexia with consideration to the influence of intra-day variation of metabolite levels.

Metabolomics analysis identified some metabolites whose levels differed significantly in patients with cachexia from levels in those without. While speculating as to why levels of a substantial number of metabolites differed markedly by time of sample collection is difficult in the present study, we did find that levels of paraxanthine remained lower in the cohort with cachexia than in that without throughout the day. Paraxanthine, the dimethyl derivative of xanthine, is the preferential path of caffeine metabolism in humans. Paraxanthine cannot be synthesized as a natural plant product and is seen only as a natural metabolite of caffeine in animals. Because paraxanthine, like caffeine, is a psychoactive central nervous system stimulant and increases energy expenditure and lipid turnover, it may be related to the pathogenesis of cancer cachexia or anorexia [22], [23]. It remains unclear that the reduction in the levels of paraxanthine is associated with regulation in order to maintain homeostasis of lipid mobilization.

While paraxanthine concentrations were not detectable using the common HPLC assay under fasting conditions in a previous study [23], [24], we were able to measure values using a GCMS-QP2010 Ultra in the present study. We did not strictly control caffeine intake in subjects but recognized on post-hoc survey that one patient with outlying values in the cohort with cachexia was a heavy drinker of coffee. The association between paraxanthine level and cachexia should be confirmed in further validation studies.

In agreement with the results of previous studies [9], [21], [25], [26], median IL-6 and TNF-α levels in the cohort with cachexia were nearly double those in the cohort without, and leptin levels in the cohort with cachexia were around half those in the cohort without, albeit without statistical significance. Serum leptin levels in healthy volunteers vary substantially by gender (women tend to have higher values than men), age (elderly tend to have higher values than younger individuals), nutritional status, and body mass index [21]. Wallace et al. demonstrated a significant positive correlation between body fat loss and increase in leptin levels of healthy subjects and cancer patients (r = 0.731) [27]. The large inter- and intra-individual variation in levels in the present study suggests that, although leptin is indeed involved in the pathophysiology of cachexia, it cannot serve as a useful marker at one time. Intra-day variation in leptin suggests that leptin level is associated with anorexia and eating behavior in patients with cancer cachexia, as well as amount of body fat [28], [29].

Several pharmacological and nutritional approaches to the treatment of cancer cachexia have been evaluated. When nutritional strategies alone were found insufficient for improving cachectic syndrome, pharmacological approaches, such as steroids, methylprogesterone, and ghrelin agonists, to counteract metabolic changes were tried, albeit without success [8]. Development of a successful treatment method will likely require a better understanding of the pathogenesis of cancer cachexia and identification of a dynamic metabolic surrogate marker of pharmacological intervention.

We explored the intra-day variation in metabolites in cancer cachexia by means of metabolomics. Although a major limitation of this study is the small sample size of cachectic patients with pancreatic cancer, a cohort which included only one female, prominent intra-day variance in serum metabolite levels was demonstrated in pancreatic cancer patients, regardless of cachexia. Metabolomics may be a useful tool in identifying the dynamic metabolic response. However, many metabolites identified by metabolomics showed large intra- and inter-day variations in levels. We therefore believe that metabolomics should be evaluated while taking into account those variations [30]. Although the level of paraxanthine remained significantly lower in the cohort with cachexia throughout the day unrelated to the intra-day variation, we consider that a time-matched method may be necessary to minimize influence of intra-day variation of values due to circadian rhythm in metabolomic research. These findings should be confirmed in further validation studies. The identification of metabolites involved in cancer cachexia will leads to the elucidation of its pathophysiology.

In conclusion, we demonstrated here that levels of some serological markers and metabolites were affected by cancer cachexia. We found that many metabolites exhibited substantial intra- and inter-day variation, suggesting the potential need to pay attention to intra-day variation in these levels in metabolomics research.

## Supporting Information

Table S1 **List of metabolites detected in subjects' serum using our GC/MS-based metabolomics analysis system.**
(DOCX)

Checklist S1 **TREND Statement Checklist.**
(PDF)

Protocol S1 **Clinical Study Protocol (Japanese version).**
(DOC)

Protocol S2 **Clinical Study Protocol (English version).**
(DOC)

Statement from Their Ethics Committee or Institutional Review Board S1 (PDF)
