# Supplementary material for: Metabolomics Evaluation of Serum Markers for Cachexia and Their Intra-Day Variation in Patients with Advanced Pancreatic Cancer
Source: PLoS One. 2014 Nov 20;9(11):e113259. doi: 10.1371/journal.pone.0113259 (PMC4239056; doi:10.1371/journal.pone.0113259)
Supplement: Table S1 — List of metabolites detected in subjects' serum using our GC/MS-based metabolomics analysis system. (DOCX) [file pone.0113259.s001.docx]

Supplemental data

Table S1

List of metabolites which were detected in the subjects’ serum in our GC/MS-based metabolomics analysis system.

| 2-Hydroxypyridine | Glyceraldehyde_2 | Methylsuccinic acid | Malic acid | β-Glutamic acid | 1,6-Anhydroglucose | Hypoxanthine | 1-Hexadecanol | Tryptophan |
| --- | --- | --- | --- | --- | --- | --- | --- | --- |
| Pyruvate+Oxalacetic acid | Valine(2TMS) | Uracil | Threitol | Glutamic acid | Arabitol | Citrulline | ParaXanthine | Spermidine |
| Lactic acid | Oxamic acid | Fumaric acid | meso-erythritol | Phenylalanine | Ribitol | Dimethylbenzimidazole | N-α-Acetyl-L-Ornithine_2 | Cysteine+Cystine |
| Glycolic acid | 2-Aminoethanol | Serine(3TMS) | Adipic acid | p-Hydroxybenzoic acid | Glycerol-2-Phosphate | 1,5-Anhydro-D-glucitol | Glucarate | ɤ-Glutamyl cysteine |
| Isobutylamine | n-Caprylic acid | Nonanoic acid(C9) | Acetylsalicylic acid | Xylose_2 | N-Acetyl-L-Glutamate_1 | Tagatose_2(or Psicose_2) | S-Benzyl-L-Cysteine_1 | 3-Hydroxy-DL-Kynurenine |
| Alanine(2TMS) | Glycerol | N-Acetyl-DL-Valine | Aspartic acid | 4-Hydroxyphenylacetic acid | Glutamine | α-Sorbopyranose_1(or Fructose_1) | Palmitoleate | 2'-Deoxyuridine_2 |
| Ketovaline_1 | Phosphate | N-FormylGlycine | Methionine | Lyxose_2 | O-Phosphoethanolamine | Mannose_1 | N-Acetyl-D-Glucosamine_2 | Uridine_2 |
| HydroxyButyrate | Isoleucine | Threonine(3TMS) | trans-4-Hydroxy-L-proline | Threo-β-HydroxyAspartic acid | 2-Deoxy-D-glucose | Allantoin_1 | Dopamine | Biotin |
| Oxalate | Proline | 2,3-Bisphospho-glycerate | Pyroglutamic acid | Lauric acid | Benzen-1,4-Dicarboxylic acid | Glucose_2 | Inositol | Inosine |
| 3-Hydroxy-Butyrate | Maleic acid | Glutaric acid | 2-Thiouracil | N-Acetyl-L-Aspartic acid_2 | Shikimic acid | Lysine(4TMS) | Dopa | Sucrose |
| 4-Hydroxypyridine | Glycine(3TMS) | β-Alanine | Pyrogallol | Asparagine | 2-Aminopimelic acid | Galactosamine_1 | DethioBiotin | Trehalose |
| 2-Aminobutyric acid | Succinic acid(or aldehyde) | Phenoxyacetic acid | Creatinine | Taurine | Glycyl-Glycine_1 | Glucuronate_1 | Kynurenine | Melibiose_1 |
| Ketoisoleucine_1 | Catechol | Prolinamide | Acetoacetic acid | Cysteine Sulfonic acid | Citric acid + Isocitric acid | Tyrosine | Cystathionine |  |
| 3-Hydroxyisovaleric acid | Glyceric acid | homoserine | 2-Isopropylmalic acid | Xylitol | Ornithine | Coniferyl aldehyde_1 | 5-Hydroxyindoleacetate |  |
