# Supplementary material for: Metabolomics Evaluation of Serum Markers for Cachexia and Their Intra-Day Variation in Patients with Advanced Pancreatic Cancer
Source: PLoS One. 2014 Nov 20;9(11):e113259. doi: 10.1371/journal.pone.0113259 (PMC4239056; doi:10.1371/journal.pone.0113259)
Supplement: Checklist S1 — TREND Statement Checklist. (PDF) [file pone.0113259.s002.pdf]

TREND Statement Checklist

Paper Section/ Topic	Item No	Descriptor	Reported? ✔	Pg #
**Title and Abstract**				
Title and Abstract	1	Information on how unit were allocated to interventions	✔	Abstract
		Structured abstract recommended	✔	Abstract
		Information on target population or study sample	✔	Abstract
**Introduction**				
Background	2	Scientific background and explanation of rationale	✔	Introduction
		Theories used in designing behavioral interventions	✔	Introduction
**Methods**				
Participants	3	Eligibility criteria for participants, including criteria at different levels in recruitment/sampling plan (e.g., cities, clinics, subjects)	✔	Method-Patient selection criteria
		Method of recruitment (e.g., referral, self-selection), including the sampling method if a systematic sampling plan was implemented	✔	Method-Patient selection criteria
		Recruitment setting	✔	Method-Patient selection criteria
		Settings and locations where the data were collected	✔	Method-Patient selection criteria
Interventions	4	Details of the interventions intended for each study condition and how and when they were actually administered, specifically including:	✔	Method-Serum collection and preparation
		○ Content: what was given?	✔	Method-Serum collection and preparation
		○ Delivery method: how was the content given?	✔	Method-Serum collection and preparation
		○ Unit of delivery: how were the subjects grouped during delivery?	✔	Method-Serum collection and preparation
		○ Deliverer: who delivered the intervention?	✔	Method-Serum collection and preparation
		○ Setting: where was the intervention delivered?	✔	Method-Serum collection and preparation
		○ Exposure quantity and duration: how many sessions or episodes or events were intended to be delivered? How long were they intended to last?	✔	Method-Serum collection and preparation
		○ Time span: how long was it intended to take to deliver the intervention to each unit?	✔	Method-Serum collection and preparation
		○ Activities to increase compliance or adherence (e.g., incentives)	✔	Method-Serum collection and preparation
Objectives	5	Specific objectives and hypotheses	✔	Method-Objective and outcome
Outcomes	6	Clearly defined primary and secondary outcome measures	✔	Method-Objective and outcome
		Methods used to collect data and any methods used to enhance the quality of measurements	✔	Method-Objective and outcome
		Information on validated instruments such as psychometric and biometric properties	✔	Method-Objective and outcome
Sample Size	7	How sample size was determined and, when applicable, explanation of any interim analyses and stopping rules	✔	Method-Statistical analysis
Assignment Method	8	Unit of assignment (the unit being assigned to study condition, e.g., individual, group, community)	✔	Method-Patient selection criteria
		Method used to assign units to study conditions, including details of any restriction (e.g., blocking, stratification, minimization)	✔	Method-Patient selection criteria
		Inclusion of aspects employed to help minimize potential bias induced due to non-randomization (e.g., matching)	✔	Method-Patient selection criteria

TREND Statement Checklist

Blinding (masking)	9	Whether or not participants, those administering the interventions, and those assessing the outcomes were blinded to study condition assignment; if so, statement regarding how the blinding was accomplished and how it was assessed.	✔	Method-Objective and outcome
Unit of Analysis	10	Description of the smallest unit that is being analyzed to assess intervention effects (e.g., individual, group, or community)	✔	Method-Statistical analysis
		If the unit of analysis differs from the unit of assignment, the analytical method used to account for this (e.g., adjusting the standard error estimates by the design effect or using multilevel analysis)	✔	Method-Statistical analysis
Statistical Methods	11	Statistical methods used to compare study groups for primary methods outcome(s), including complex methods of correlated data	✔	Method-Statistical analysis
		Statistical methods used for additional analyses, such as a subgroup analyses and adjusted analysis	✔	Method-Statistical analysis
		Methods for imputing missing data, if used	✔	Method-Statistical analysis
		Statistical software or programs used	✔	Method-Statistical analysis
**Results**				
Participant flow	12	Flow of participants through each stage of the study: enrollment, assignment, allocation, and intervention exposure, follow-up, analysis (a diagram is strongly recommended)	✔	Result + Fig 1
		○ Enrollment: the numbers of participants screened for eligibility, found to be eligible or not eligible, declined to be enrolled, and enrolled in the study	✔	Result + Fig 1
		○ Assignment: the numbers of participants assigned to a study condition	✔	Result + Fig 1
		○ Allocation and intervention exposure: the number of participants assigned to each study condition and the number of participants who received each intervention	✔	Result + Fig 1
		○ Follow-up: the number of participants who completed the follow-up or did not complete the follow-up (i.e., lost to follow-up), by study condition	✔	Result + Fig 1
		○ Analysis: the number of participants included in or excluded from the main analysis, by study condition	✔	Result + Fig 1
		Description of protocol deviations from study as planned, along with reasons	✔	Result + Fig 1
Recruitment	13	Dates defining the periods of recruitment and follow-up	✔	Result-Patient's characteristics
Baseline Data	14	Baseline demographic and clinical characteristics of participants in each study condition	✔	Result-Patient's characteristics + Table 1
		Baseline characteristics for each study condition relevant to specific disease prevention research	✔	Result-Patient's characteristics + Table 1
		Baseline comparisons of those lost to follow-up and those retained, overall and by study condition	✔	Result-Patient's characteristics + Table 1
		Comparison between study population at baseline and target population of interest	✔	Result-Patient's characteristics + Table 1
Baseline equivalence	15	Data on study group equivalence at baseline and statistical methods used to control for baseline differences	✔	Result-Patient's characteristics + Table 1

TREND Statement Checklist

Numbers analyzed	16	Number of participants (denominator) included in each analysis for each study condition, particularly when the denominators change for different outcomes; statement of the results in absolute numbers when feasible	✔	Result-Patient's characteristics + Table 1
		Indication of whether the analysis strategy was "intention to treat" or, if not, description of how non-compliers were treated in the analyses	✔	Result-Patient's characteristics + Table 1
Outcomes and estimation	17	For each primary and secondary outcome, a summary of results for each estimation study condition, and the estimated effect size and a confidence interval to indicate the precision	✔	Result-Metabolomics
		Inclusion of null and negative findings	✔	Result-Metabolomics
		Inclusion of results from testing pre-specified causal pathways through which the intervention was intended to operate, if any	✔	Result-Metabolomics
Ancillary analyses	18	Summary of other analyses performed, including subgroup or restricted analyses, indicating which are pre-specified or exploratory	✔	Result-Metabolomics + Serological markers
Adverse events	19	Summary of all important adverse events or unintended effects in each study condition (including summary measures, effect size estimates, and confidence intervals)	✔	Result-Treatment and survival
**DISCUSSION**				
Interpretation	20	Interpretation of the results, taking into account study hypotheses, sources of potential bias, imprecision of measures, multiplicative analyses, and other limitations or weaknesses of the study	✔	Discussion
		Discussion of results taking into account the mechanism by which the intervention was intended to work (causal pathways) or alternative mechanisms or explanations	✔	Discussion
		Discussion of the success of and barriers to implementing the intervention, fidelity of implementation	✔	Discussion
		Discussion of research, programmatic, or policy implications	✔	Discussion
Generalizability	21	Generalizability (external validity) of the trial findings, taking into account the study population, the characteristics of the intervention, length of follow-up, incentives, compliance rates, specific sites/settings involved in the study, and other contextual issues	✔	Discussion
Overall Evidence	22	General interpretation of the results in the context of current evidence and current theory	✔	Discussion

From: Des Jarlais, D. C., Lyles, C., Crepaz, N., & the Trend Group (2004). Improving the reporting quality of nonrandomized evaluations of behavioral and public health interventions: The TREND statement. *American Journal of Public Health*, 94, 361-366. For more information, visit: http://www.cdc.gov/trendstatement/
