# Supplementary material for: Metabolomics Evaluation of Serum Markers for Cachexia and Their Intra-Day Variation in Patients with Advanced Pancreatic Cancer
Source: PLoS One. 2014 Nov 20;9(11):e113259. doi: 10.1371/journal.pone.0113259 (PMC4239056; doi:10.1371/journal.pone.0113259)
Supplement: Protocol S1 — Clinical Study Protocol (Japanese version). (DOC) [file pone.0113259.s003.doc]

**進行期膵臓がん患者における悪液質とそれに関わる代謝産物の日内変動に関するメタボローム解析**

**1.1. 目的**

メタボローム解析により進行期膵臓がん患者の悪液質により変化する代謝産物を探索する。

**2. 背景**

**2.1. 膵臓がんと悪液質**

現在悪性腫瘍は我が国における死亡原因の第一位であり、年々増加の一途をたどっている。中でも膵臓がんは厚生労働省による人口動態調査(2007)によると死亡者数は24,634人と癌の死因別では男女とも第5位で、年々増加傾向にある[1]。初期には無症状のことも多いが、病気の進行とともに腹痛、背部痛、閉塞性黄疸、耐糖能異常のほか食欲不振、体重減少といった悪液質の進行を認めることが多い予後不良の悪性腫瘍である。

膵臓がんの進行期あるいは末期においては悪液質と呼ばれる栄養障害により衰弱した状態（体重減少、るいそう、低タンパク血症、浮腫など）を認めることがある。これらは病状の進行に伴う炭水化物やタンパク質の代謝変化が原因と考えられている。現在IL1、IL6、TNF-α、TGF-βなどの炎症性サイトカインや血管内皮増殖因子（VEGF）の分泌が悪液質の病態に関わるとの報告もあるが、実際に悪液質の際にどのような変化が全身で起こっているかは不明である[2, 3]。

悪液質の病態を解明するためには、がんに罹患した患者において、悪液質を有する群および悪液質を有さない患者群における病態変化の比較検討が必要と考えられる。また生物には日内変動（サーカディアンリズム）とよばれる1日のサイクルにより睡眠、覚醒、血圧、ホルモン分泌などが変動することが知られているため、悪液質における代謝変化の検討を行うためには日内変動による影響も検討する必要があると考えられる[4, 5]。

**2.2. メタボローム解析とは**

生命の設計図は遺伝子で構成されているが、環境的要因などの影響もあり、実際には多くのタンパク質や代謝産物がその生命活動を担っている。そのため、がんや糖尿病など各種疾患におけるタンパク質や代謝産物の変動は遺伝子の発現量よりも、ダイナミックである可能性があると考えられている。この生物によって生み出された代謝産物を網羅的に解析することを「メタボローム解析」という。各種疾患の発症による一連の代謝産物の変動をパターン解析する、すなわち各疾患に対して単独ではなく複数のバイオマーカーを評価対象とする研究を行うことで、新たな疾患の病態解明が可能となると期待されている。

一般に研究のブレイクスルーとしては、血清（あるいは、血漿）、尿、呼気濃縮液、組織、糞便など生体試料から、水溶性代謝産物と脂溶性代謝産物をそれぞれ抽出し、そこに含まれる質量数1,000以下の代謝産物が液体クロマトグラフ質量分析計、ならびにガスクロマトグラフ質量分析計により測定される。得られたデータをマインニングし、主成分分析を実施した結果、各疾患に、さらには疾患の進行度、悪性度それぞれに特異的な代謝産物の発現パターンを決定し（メタボリックフィンガープリンティング）、それを病態診断に活用することが可能となる。さらに、この結果を基に、実際にしている代謝産物を同定し（メタボライトプロファイリング）、それをバイオマーカーとした早期診断システムを確立することが期待される。水溶性代謝産物中には、糖や有機酸、アミノ酸などが、脂質代謝産物中には、脂肪酸やコレステロールなどが存在し、それらは、エネルギー代謝や細胞増殖など様々な生体機能に重要な役割を担っている。現在、疾患バイオマーカーのほとんどがタンパク質であるが、実際に生体機能を直接的に制御しているのは、タンパク質の作用により変動した低分子代謝産物であることが多いことから、それら複数の代謝産物をバイオマーカーとして使用することは、早期診断、さらには、様々な病態をトレースする上で非常に有用であると考えられる。また、代謝産物の変動を捉えることは、未知の病態の解明につながることも予想される。

**2.3. 研究方法**

**2.3.1. 研究対象**

悪液質を有さない進行期膵臓がん患者10人、および悪液質を有する進行期膵臓がん患者10人を対象とする。

（本試験は探索的であり結果において、より有用な解析方法が見つかった際には解析方法を変更する可能性があり、それに伴い必要症例数を追加する可能性がある。）

**2.3.2. 研究方法**

　対象患者より血清を①朝食前6:30、②昼食前11:30、③夕食前16:30、④眠前21:30の4ポイント採取する。

**2.4. 本試験の意義**

本試験は悪液質を伴わない進行期膵臓がん患者および悪液質を伴う進行期膵臓がん患者の血清からメタボローム解析を行う。その群間における代謝パターンの違いおよび代謝産物の日内変動の変化を測定することで、悪液質の病態に伴う代謝変化を解析し、関連する代謝産物を同定することで悪液質の病態を探索することを目的とする。また血液よりDNAサンプルを抽出し、既知もしくは本試験によって得られた悪液質に関わる代謝産物に関連する遺伝子の変異を検討する。本試験により悪液質に関わる特定の代謝産物や代謝変化が同定できた場合には、将来の悪液質を有する患者の治療に貢献する可能性があると考えられる。

**3. 患者登録基準**

適格基準のすべてを満たし、除外基準のいずれにも該当しない患者を適格とする。同一の患者が悪液質を有さない膵臓がん患者群と悪液質を有する膵臓がん患者群の両群に、経過を経て参加することを許容するが、その場合には再度同意を取得する。

**3.1. 患者適格基準**

①組織学的、細胞診学的および画像診断において膵臓がん（Adenocarcinoma, adenosquamous carcinoma）と診断された患者

（画像診断にのみであっても患者登録は可能とするが、より診断精度の高い方法により膵臓がんの診断が否定されれば解析対象からは除外する。）

②局所進行もしくは転移性膵臓がん患者（UICC TNM分類にてStage IIB-IV）

③１．悪液質を有さない膵臓がん患者：PS 0-2、Alb 3.5mg/dL以上、食欲不振Gr0-1(NCI-CTC ver.3)、6か月以内の体重減少なし（5%未満）、6か月程度の予後が見込める患者

　２．悪液質を有する膵臓がん患者：PS 1-4、食欲不振Gr1-4(NCI-CTC ver.3)、6か月以内に体重減少(10%以上)、6か月程度の予後の見込みが困難な患者

④20歳以上の患者

⑤主要な臓器機能が保持されている患者

1. 肝機能

総ビリルビン：実施医療機関における基準値上限の1.5倍以下

AST, ALT：実施医療機関における基準値上限の2.5倍以下

1. 腎機能

血清クレアチニン：実施医療機関における基準値上限の1.5倍以下

⑥本試験への参加について文書による同意が得られた患者

**3.2. 患者除外基準**

①切除可能膵臓がんの患者

②膵原発神経内分泌腫瘍の患者

③1週間以内に化学療法を受けた患者

④1か月以内に全身麻酔を要する外科的手術を受けた患者

⑤1か月以内に膵臓がんに対する根治的放射線治療を受けた患者

⑥2週間以内に症状緩和を目的とした放射線治療を受けた患者

⑦臨床上問題となる感染症を有する患者

⑧コントロール困難な糖尿病を有する、またはHbA1c > 8%の患者

⑨ステロイド（プレドニゾロン換算で10mg/day以上）の投与を受けている患者

⑩活動性の重複がんを有する患者

⑪その他、試験責任医師または試験分担医師が本試験の対象として適当でないと判断した患者

**4. 登録方法**

**4.1. 登録手順**

１）研究分担者は対象患者が適格基準をすべて満たし、除外基準のいずれにも該当しないことを確認し、症例登録票に必要事項をすべて記入の上、研究事務局に連絡をする。

２）研究事務局は症例登録票の内容を確認し、不備があった場合はその内容を研究分担者に確認の上、すべての基準が満たされていることを確認する。

３）登録日は登録手続きが完了した日とする。

４）研究事務局で症例登録票を保管する。

５）研究分担者は登録されていることを確認後、検体採取を行う。

**5. 研究方法**

**5.1. 検体の採取**

**5.1.1. メタボローム解析用検体の採取**

１）対象患者より血清採取のために5mlの血液を次のポイントで採取する。

２）①朝食前6:30、②昼食前11:30、③夕食前16:30、④眠前21:30の4ポイントで採血をする。（食事の摂取は問わない）

３）各医療機関において血清分離を行い検体保存用チューブに0.5mlずつ4本に分注する。

**5.2.　DNA保存用検体の採取**

**5.2.1.　末梢血単核球の採取**

末梢血（全血で約10ml）を採取する。採血後すみやかに単核球を分離し、凍結保存する。

**5.2.2.　DNA保存検体の廃棄方法**

血液から抽出したDNAサンプルは保存され、本試験における解析に用いられる。解析後の残余試料は本試験が完了し結果が報告された時点で、ほかのすべての残余試料とともに熱処理など適切な方法で廃棄される。

**5.3. 検体の保管**

各医療機関において-80度のDeep freezerにて、測定までの間保存を行う。

**5.4. 検体の測定**

神戸大学医学部質量分析総合センターにおいてメタボローム解析を行う。

**5.5. 検体の匿名化**

検体は登録された時点で、個人情報管理者の管理の下で管理され、匿名化を行う。具体的には、個人情報の管理は個人情報管理者により、個人情報管理専用のコンピュータによりパスワード、暗号化を用いて管理され、個人情報および症例・符号対照表には個人情報管理者または個人情報分担管理者以外のアクセスができないようにする。匿名化については症例登録時に検体に対応する個人識別情報は検体組織部位、症例番号、採取時期を示す6文字の英数字に変換し、以後、検体は符号化された番号のみで取り扱い、解析に用いる。

**5.6. QOL調査**

採血当日もしくは採血前72時間以内にQLQ-C30にて行う。

**5.7. メタボローム解析**

**5.7.1　使用機器**

液体クロマトグラフ質量分析計　LCMS-IT-TOF（株式会社 島津製作所）

ガスクロマトグラフ質量分析計　GCMS-2010（株式会社 島津製作所）

**5.7.2　液体クロマトグラフ質量分析計を用いたメタボローム解析**

**サンプル前処理　血清（あるいは、血漿）中の水溶性代謝産物の測定**

1.5ml容エッペンドルフチューブに血清（あるいは、血漿）10 μlと50 μg/mlの2-isopropylmalic acidなどの内部標準を10 μl添加する。続いて、メタノール0.5mlを添加して混合する。次に、蒸留水0.25 mlを添加して混合する。さらに、クロロホルム0.5 mlを添加して混合する。続いて、1200 rpmで混合しながら37℃で30分間インキュベートする。溶液を15000 rpm、4℃で5分間遠心分離し、その上清200 μlを新しい1.5ml容エッペンドルフチューブに回収し、スピードバック遠心濃縮装置により濃縮する。その後、凍結乾燥機により溶液を乾固させる。乾固物を5％アセトニトリル溶液75 μlに再溶解させ、0.22 μmフィルタースピンカラムよりフィルター処理し、回収された溶液15 μlをLCMS-IT-TOFによる測定に供する。

**データ処理**

LCMS-IT-TOFの測定により得られたマスクロマトグラムをデータ処理ソフトIonXtractViewに供することでデータピッキングを行い、ピーク強度とm/zの情報を数値化する。内部標準である2-isopropylmalic acid（m/z；175.01）のピーク強度を基に各サンプル間のデータを標準化する。

**主成分分析**

標準化したデータを多変量解析ソフトPirouette、あるいはSimca-Pに供することで主成分分析を実施し、Scoreの結果により疾患の有無や悪性度、進行度などを分類・判別できるか否かを検討する。分類・判別が可能となれば、各主成分に対するLoadingのデータを基に、MS^n^解析、データベース解析、ライブラリー解析を駆使して、実際に変動が見られた水溶性代謝産物を同定する。

**5.7.3　ガスクロマトグラフ質量分析計を用いたメタボローム解析**

**サンプル前処理　血清（あるいは、血漿）中の水溶性代謝産物の測定**

1.5ml容エッペンドルフチューブに血清（あるいは、血漿）10 μlと混合溶媒（メタノール：クロロホルム：水＝2.5：1：1）50 μlとを添加し混合する。次に、1 mg/mlの2-isopropylmalic acid（内部標準）を10 μl添加して混合する。続いて、1200 rpmで混合しながら37℃で30分間インキュベートする。溶液を15000 rpm、4℃で3分間遠心分離し、その上清45 μlを新しい1.5ml容エッペンドルフチューブに回収する。そこに、40 μlの超純水を添加し、混合した後に15000 rpm、4℃で3分間遠心分離する。その上清50 μlを新しい1.5ml容エッペンドルフチューブに回収し、スピードバック遠心濃縮装置により濃縮する。その後、凍結乾燥機により溶液を乾固させる。乾固物を20 mg/ml methoxyamine pyridine溶液20 μlに溶解し、20分間水浴超音波処理を行う。続いて、1200 rpmで混合しながら30℃で90分間インキュベートし、そこにMSTFAを10 μl添加する。37℃で30分間インキュベート後、15000 rpm、4℃で3分間遠心分離し、その上清をGCMS-2010による測定に供する。

**GCMS-2010による測定**

TMS化したサンプルのGCMS-2010による測定は、GCMSsolutionのOA_TMS.qgmメッドファイルを使用する。カラムはAiglent J&W社製のDB-5（30 m x0.25 mmID df1.00μm）を使用する。

**データ処理**

GCMS-2010の測定により得られたTICクロマトグラムのファイル形式をAIA形式に変換し、データ処理ソフトPiroTrans135により内部標準を用いた保持時間方向（X軸）補正、ならびにピーク強度方向（Y軸）補正を行う。次に、データ処理ソフトLineUPを用いて保持時間方向（X軸）補正を行う。続いて、データ処理ソフトPiroTrans135により、ベースライン補正を行い、ファイル形式をdatファイル形式に変換する。

**主成分分析**

標準化したデータ（datファイル）を多変量解析ソフトPirouette、あるいはSimca-Pに供することで主成分分析を実施し、Scoreの結果により疾患の有無や悪性度、進行度などを分類・判別できるか否かを検討する。分類・判別が可能となれば、各主成分に対するLoadingのデータを基に、データベース解析とライブラリー解析により、実際に変動が見られた水溶性代謝産物、アミノ酸、そして、脂肪酸を同定する。

**6. 評価項目**

**6.1. 患者評価項目**

１）患者背景：性別、登録時年齢、生年月日、患者識別番号

２）一般所見：PS、身長、体重、体温、脈拍、血圧

３）腫瘍所見：病理学的、細胞診学的確定診断の有無、病期（TNM分類）、腫瘍マーカー（CEA、CA19-9、DUPAN-2など）

４）血液学的検査所見：WBC、ANC、Hb、Hct、Plt、TP、Alb、T-Bil、AST、ALT、LDH、アミラーゼ、リパーゼ、エラスターゼ、BUN、Cr、総コレステロール、LDL、HDL、TG、CRP、Glu、HbA1c

５）服薬状況

**6.2. DNA保存用検体**

血液より抽出したDNAサンプルは、既知もしくは本試験によって得られた悪液質に関わる代謝産物に関連する遺伝子の変異を検出するためにのみ用いる。探索的研究であるため現時点でDNA解析部位は不明であるが、DNA解析を施行する際には明確な遺伝子解析部位に対して再度倫理委員会に申請を行い、承認を必要とする。ただし被験者に対する再同意は必要としない。また本試験の結果によって遺伝子解析の実施の必要がないと判断された場合には、解析を実施しないこともある。

検体の取る扱い、匿名化については**5.研究方法**、**5.5.検体の匿名化**に記載する。

**7. エンドポイントと統計学的考察**

**7.1. エンドポイント**

Primary endpoint：メタボローム解析によって同定された悪液質に関わる代謝産物

Secondary endpoints：悪液質に関わる代謝産物の日内変動。悪液質に関わると考えられているIL-6、TNF-α、VEGFなどの炎症性サイトカイン、レプチンなど血清学的マーカーの悪液質の有無による変化。悪液質の有無とQOL調査の変化。

**7.2. 登録数設定根拠**

本試験は探索的であり統計学的設定根拠は無いが、過去に行われたメタボローム解析研究においては各群10例程度で有意な結果が導かれているため、本試験でも各群10例とした。ただし本試験の結果においてより有用な解析方法が見つかった際には解析方法を変更し、それに伴い必要症例数を追加することがありうる。

**8. 研究機関**

神戸大学医学部付属病院腫瘍内科、神戸大学医学研究科質量分析総合センター

**9. 患者登録期間**

神戸大学医学研究科長承認年月日から2012年9月まで3年間

**10. 試験の安全性の確保**

**10.1. 被験者の安全性を確保するための基本的事項**

試験責任（担当）医師は、被験者の試験参加中、必要かつ適切な観察・検査を行い、被験者の安全性確保に留意する。有害事象の発現に際しては、必要に応じて適切な処置を施し、被験者の安全性確保に留意するとともに、その原因究明に努める。

**10.2. 予想される有害事象**

採血5ml/回、4回、DNA保存用採血10mlの合計30mlの血液採取において患者に対する健康上の悪影響はないと考えられる。

**11. 倫理的事項**

**11.1. 患者の保護**

本試験を実施するにあたり、「ヘルシンキ宣言」（2002年 米国ワシントン）および「臨床研究に関する倫理指針」（2008年7月31日 厚生労働省）の倫理的原則を遵守して、患者の人権、福祉および安全を最大限に確保することとする。本試験に関する、有害事象、研究結果その他関連データを報告する場合には、患者の身元の秘密を保全し、人権保護について十分配慮することとする。

**11.2. 患者への説明同意**

**11.2.1. 患者の同意**

担当医は、登録前に同意説明文書・同意書に基づき、事前に本研究の意義、目的、方法、予測される結果や不利益について検体提供者に説明し、文書により自由意思による検体提供者の同意を得る。担当医並びに検体提供者は、同意書に署名及び日付（説明日、同意取得日）を記載する。同意説明文書・同意書（写）を検体提供者に渡すとともに、原本をカルテ内に保管する。

**11.2.2. 同意説明文書・同意書による検体提供者への説明事項**

同意取得に際し、下記説明事項を同意説明文書・同意書を用いて説明する。

- - - - 研究概要
      - 研究目的
      - 研究方法
      - 参加予定期間
      - 参加予定患者数
      - 研究期間中及び終了後の試(資)料等の取り扱いの方針
      - 関連する遺伝子の変異解析について
      - 本試験の実施機関
      - 予想される臨床上の利益(効果)及び不利益(副作用)
      - 研究協力の任意性
      - 研究協力の撤回の自由
      - 新しい重大な情報の開示
      - プライバシーの保護
      - 研究成果の公表
      - 研究から生じる知的財産権の帰属
      - 費用負担に関する事項
      - 謝礼の有無
      - 研究計画書等の開示
      - 守らなければならない事項
      - 研究責任者・研究協力者の連絡先
      - 相談窓口

**11.3. 患者の同意**

試験についての説明後、患者が試験の内容をよく理解したことを確認した上で、試験への参加について依頼する。本試験への参加については患者の完全な自由意思によるものとし、患者本人が試験参加に同意した場合、同意を得た日付を記載し患者本人および説明を行った医師が署名する。同意書は、1部は患者本人に手渡し、1部はカルテに保管する。

**11.4. 研究結果の開示**

　　本研究の研究結果により被検者の治療や医学上の利益に重大な影響を与えると判明した場合には、再度説明を行なう。

**11.5. 研究計画書の開示**

　　被検者もしくはその関係者が本研究の実施計画書の開示を希望される場合は、必要に応じて開示を行なう。

**11.6. 検体の保存・管理**

採取した検体は速やかに研究施設内の冷凍庫に保存され、厳重に管理される。

**11.7. 個人識別情報の管理**

提供を受けた検体は、匿名化担当者が検体識別番号により符号化する。提供者と符号の対照表は匿名化担当者により厳重に保管される。

**11.8. 施設内倫理審査委員会などでの承認**

本試験はすでに神戸大学医学部倫理委員会において承認を受けている。

**12. 研究結果の公表**

　研究代表者が共同研究者と協議の上、研究代表者、共同研究者、または研究協力者が論文、学会発表を行う。

**13.　研究費**

　一般診療および通常臨床検査に関わる費用は通常の医療保険制度に沿った患者負担とする。メタボローム解析に関する費用、保険外採血検査に関しては神戸大学医学部腫瘍内科において負担する。

**14.　健康被害に関する補償**

　本試験に起因した健康被害に生じた場合には、通常の保険医療に基づいた適切な医療を提供する。補償は医師に過失がない限り行わない。

**15.　知的財産権の帰属**

　本研究から生じる知的財産権は神戸大学または研究者に帰属する。

**16. 研究代表者、試験分担者、個人情報分担管理者**

研究代表者：南 博信　（神戸大学医学部付属病院：腫瘍内科教授）

神戸市中央区楠町7丁目５－１

TEL:　078-382-5820　　FAX:　078-382-5821

研究分担者：向原 徹　（神戸大学医学部附属病院：腫瘍内科准教授）

　　　　　 清田 尚臣 （神戸大学医学部附属病院：腫瘍内科助教）

藤原 豊 　（神戸大学医学部附属病院：腫瘍内科助教）

　　　　　　茶屋原 菜穂子（神戸大学医学部附属病院：腫瘍内科医員）

　　　　　　富岡 秀夫（神戸大学医学部附属病院：腫瘍内科医員）

　　　　　　船越 洋平（神戸大学医学部附属病院：腫瘍内科医員）

吉田　優（神戸大学医学研究科質量分析総合センター特命准教授）

　　　　　　西海　信（神戸大学医学研究科質量分析総合センターCOE研究員）

東　健　（神戸大学医学部附属病院：消化器内科教授）

　　　　　　久津見 弘（神戸大学医学部附属病院：消化器内科特命教授）

　　　　　　具　英成（神戸大学医学部附属病院：肝胆膵外科教授）

　　　　　　松本逸平（神戸大学医学部附属病院：肝胆膵外科助教）

個人情報管理者　：

　　　　　　前田英一（神戸大学医学部附属病院：医療情報部教授）

個人情報分担管理補助者：

　　　　　　若宮浩子（神戸大学医学部付属病院：腫瘍内科技術補佐員）

**17. 研究事務局**

神戸大学医学部付属病院　腫瘍内科特定助教 　藤原　豊

神戸市中央区楠町7丁目５－１

TEL:　078-382-5825　　FAX:　078-382-5821

**18.　参考文献**

1 人口動態調査 厚: 2007

2 Uomo G, Gallucci F, Rabitti PG: Anorexia-cachexia syndrome in pancreatic cancer: Recent development in research and management. JOP 2006;7:157-162.

3 Tisdale MJ: Mechanisms of cancer cachexia. Physiol Rev 2009;89:381-410.

4 Hastings M, O'Neill JS, Maywood ES: Circadian clocks: Regulators of endocrine and metabolic rhythms. J Endocrinol 2007;195:187-198.

5 Liu AC, Lewis WG, Kay SA: Mammalian circadian signaling networks and therapeutic targets. Nat Chem Biol 2007;3:630-639.
