# Supplementary material for: Metabolomics Evaluation of Serum Markers for Cachexia and Their Intra-Day Variation in Patients with Advanced Pancreatic Cancer
Source: PLoS One. 2014 Nov 20;9(11):e113259. doi: 10.1371/journal.pone.0113259 (PMC4239056; doi:10.1371/journal.pone.0113259)
Supplement: Protocol S2 — Clinical Study Protocol (English version). (DOC) [file pone.0113259.s004.doc]

**Metabolomics evaluation of serum markers for cachexia and their circadian change in patients with advanced pancreatic cancer**

**Clinical Study Protocol**

**Principle investigator: Prof. Hironobu Minami**

Division of Medical Oncology/Hematology, Kobe University Graduate School of Medicine

7-5-2 Kusunoki-cho, Chuo-ku, Kobe, 650-0017, JAPAN

Phone: +81-78-382-5820　　　FAX: +81-78-382-5821

E-mail: hminami@med.kobe-u.ac.jp

**Research secretariat: Yutaka Fujiwara**

Division of Medical Oncology/Hematology, Kobe University Graduate School of Medicine

**1. Study Purpose**

To explore the change in serum metabolites of the pancreatic cancer patients with cachexia and without cachexia

**2. Background**

**2.1. Pancreatic cancer and cachexia**

Pancreatic cancer is the leading cause of cancer-related death. At the time of diagnosis, tumor resection with curative intent is only possible in 10%to 15% of subjects, leaving a large population with poor prognosis and limited therapeutic options. Overall, the 5-year survival rate is only about 4%.

Some patients with advanced or end-stage pancreatic cancer experience debilitating symptoms such as weight loss, emaciation, hypoproteinemia, and edema, also known as cachexia. These symptoms may be caused by changes in carbohydrate/protein metabolism experienced as the disease progresses. While secretion of inflammatory cytokines (IL1, IL6, TNF-α, TGF-β, etc.) and vascular endothelial growth factors (VEGFs) has been reported to be involved in the pathophysiology of cachexia, the precise mechanism of cachexia are unknown [1, 2].

Clarification of the mechanism of cachexia may require comparison of pathophysiological changes between cancer patients with and without cachexia. In addition, the physiology of living organisms is known to be affected by circadian rhythms. We think that effects of circadian rhythms may need to be considered in the evaluation of metabolic changes associated with cachexia [3, 4].

**2.2. Metabolomics**

While genes are commonly considered the “blueprint of life,” in actuality, many proteins and metabolites play essential roles in organic activities. An exhaustive analysis of metabolites produced by living organisms is called metabolomics. This analysis, which involves a pattern analysis of a series of changes in metabolites associated with the development of a certain disease, assesses multiple biomarkers rather than a single one for the disease and is therefore expected to help elucidate unknown disease pathologies.

Briefly, water-soluble and lipid-soluble metabolites are separately extracted from biological specimens such as serum (or plasma), urine, exhaled breath condensate, tissues, and feces, and metabolites with a mass number of 1,000 or less are measured using liquid chromatography/mass spectrometry and gas chromatography/mass spectrometry. After data mining, a principal component analysis as metabolic fingerprinting is performed to determine the pattern of metabolite production specific to the disease itself, stage of progression, and malignancy grade, parameters which can be used to diagnose pathological conditions. This information is also expected to be useful in identifying individual metabolites as metabolite profiling and establishing an early diagnosis system using these metabolites as biomarkers.

Most currently used disease-specific biomarkers are proteins. However, since these vital functions are often directly controlled by low-molecular metabolites affected by those proteins, these metabolites in conducting early diagnosis and in tracing the pathology of various diseases may be useful as biomarker.

**3. Methods**

**3.1. Subjects**

**T**he pancreatic cancer patients with cachexia (n=10) and without cachexia (n=10)

- Control : PS 0-2, Alb ≧ 3.5mg/dL, Anorexia Gr 0-1, Weight loss < 5% over past 6 months
- Cachexia : PS 1-4, Anorexia Gr1-4, Weight loss > 10% over past 6 months

**3.2. Significance of the study**

We investigated the difference in serum metabolites of pancreatic cancer patients with and without cachexia and analyzed the pattern and intra-day variation in metabolites using metabolomics. Our findings here may help improve treatment of patients with cachexia if specific metabolites or metabolic changes involved in cachexia can be identified.

**3.3 Criteria for subject enrollment**

**3.3.1. Eligibility criteria**

1) Patients histologically confirmed adenocarcinoma or adenosquamous carcinoma of the pancreas.

2) Locally advancer or metastatic pancreatic cancer (UICCC TNM classification Stage IVA-IVB).

3) (a) Control: PS 0-2, Alb ≧ 3.5mg/dL, Anorexia Gr 0-1 (NCI-CTC ver.3), Weight loss < 5% over past 6 months

(b) Cachexia: PS 1-4, Anorexia Gr1-4, Weight loss > 10% over past 6 months

4) Patients 20 years of age or older.

5) Patients with adequate organ function as follows:

Liver function: AST and ALT less than 2.5 times of ULN.

Total bilirubin value less than 1.5 times of ULN.

Renal function: Serum creatinine level less than 1.5 times of ULN.

6) Patients from whom written informed consent for participation in the study is obtained.

**3.3.2. Exclusion criteria**

1) Surgically resectable pancreatic cancer.

2) Patients with pancreatic neuroendocrine tumor.

3) Patients treated with chemotherapy within 7 days.

4) Patients undergone surgery within 28 days.

5) Patients treated with definitive irradiation within 28 days or palliative irradiation within 14 days.

6) Patients with active multiple primary cancer.

7) Patients with serious pre-existing medical condition such as uncontrolled infection, diabetic mellitus (ex. HbA1c level greater than 8.0%).

8) Patients taking concomitant steroids.

9) In addition, patients who the investigator determines to be unsuitable for the study.

**3.4. Collection of samples**

**3.4.1. Serum samples for metabolomics and serum markers**

Blood samples will be collected (approximately 5 ml of whole blood) at 6:30 AM, 11:30 AM, 4:30 PM, and 9:30 PM to analyze the intra-day variation. After collection of whole blood, samples were allowed to clot at room temperature, and serum was separated by centrifugation at 3,000×g for 10 min at 4 °C and stored at -80 °C until use.

**3.4.2.** **Anonymity of samples**

Samples will be managed and anonymized at the time of registration by the personal data manager. The personal data and case-coding correspondence table will be stored to ensure that only the personal data manager can access them.

**3.5. Measurement of serum markers**

Serum IL-6, TNFα, and leptin were measured by SRL Inc. (Tokyo, Japan).

**3.6. Metabolomics**

**3.6.1. Equipment**

- Liquid chromatography/mass spectrometer; LCMS-IT-TOF (Shimadzu Corporation)
- Gas chromatography/mass spectrometer; GCMS-2010 (Shimadzu Corporation)

**3.6.2. Metabolome analysis using a liquid chromatography/mass spectrometer**

**Preparing of samples; Measurement of water-soluble metabolites in serum**

Ten microliters of serum and 10 μL of internal standard (e.g. 50 μg/mL 2-isopropylmalic acid) were placed into a 1.5-mL Eppendorf tube. Methanol (0.5 mL) was then added and mixed, followed by 0.25 mL of distilled water and then 0.5 mL of chloroform. Subsequently, the mixture was incubated with rotation (1200 rpm) for 30 minutes at 37 °C. The solution was centrifuged at 15000 rpm for 5 minutes at 4 °C, and 200 μL of the supernatant was collected in another 1.5-mL Eppendorf tube and concentrated using a SpeedVac centrifugal concentrator. The solution was then lyophilized to dryness using a lyophilizer. The residue was rehydrated in 75 μL of 5% acetonitrile solution and filtered using a 0.22-μm filter spin column. Fifteen microliters of the filtrate was used for measurement with a LCMS-IT-TOF.

**Data processing**

The mass chromatogram obtained by LCMS-IT-TOF was processed using the data processing software of IonXtractView for data picking to quantify the data of peak intensity and m/z. Data of each sample were normalized based on the peak intensity of 2-isopropylmalic acid (m/z: 175.01) as an internal standard.

**3.6.3. Metabolomic analysis with the use of a gas chromatography/mass spectrometer**

**Pretreatment of samples; Measurement of water-soluble metabolites in serum (or plasma)**

Ten microliters of serum (or plasma) and 50 μL of mixed solvent (methanol:chlorofor:water = 2.5:1:1) were placed into a 1.5-mL Eppendorf tube and mixed, after which 10 μL of 1 mg/mL 2-isopropylmalic acid (internal standard) was added and mixed. Subsequently, the mixture was incubated with rotation (1200 rpm) for 30 minutes at 37 °C. The solution was centrifuged at 15000 rpm for 3 minutes at 4 °C, and 45 μL of the supernatant was collected in another 1.5-mL Eppendorf tube. Then, 40 μL of ultrapure water was added and mixed, and the mixture was centrifuged at 15000 rpm for 3 minutes at 4 °C. Fifty microliters of the supernatant was collected in a third 1.5-mL Eppendorf tube and concentrated using a SpeedVac centrifugal concentrator. The solution was then lyophilized to dryness using a lyophilizer. The residue was rehydrated in 20 μL of 20 mg/mL methoxyamine pyridine solution and ultrasonicated in a water bath for 20 minutes. Subsequently, the solution was incubated with rotation (1200 rpm) for 90 minutes at 30 °C, and 10 μL of MSTFA was added. The mixture was then incubated for a further 30 minutes at 37 °C and then centrifuged at 15000 rpm for 3 minutes at 4°C. The supernatant was used for measurement with a GCMS-2010.

**Measurement with a GCMS-2010**

GC/MS analysis was performed using a GCMS-QP2010 Ultra (Shimadzu Co., Kyoto, Japan) with a fused silica capillary column (CP-SIL 8 CB low bleed/MS; inner diameter, 30 mm × 0.25 mm; film thickness, 0.25 μm; Agilent Co., Palo Alto, CA), according to a previously described method {Tsugawa, 2011 #43}. The front inlet temperature was 230^o^C, and the flow rate of helium gas through the column was 39.0 cm/sec. The column temperature was held at 80^o^C for 2 min and then raised by 15^o^C/min to 330^o^C and held there for 6 min. The transfer line and ion-source temperatures were 250^o^C and 200^o^C, respectively. Twenty scans per second were recorded over the mass range 85-500 m/z using the Advanced Scanning Speed Protocol (ASSP, Shimadzu Co.).

**Data processing**

Data processing was performed according to the methods described in previous reports {Tsugawa, 2011 #43}{Tsugawa, 2011 #28}. Briefly, the MS data were exported in netCDF format. The peak detection and alignment were performed using the MetAlign software (Wageningen UR, The Netherlands). The resultant data were exported in CSV format and then analyzed with in-house analytical software. For semi-quantification, the peak height of each ion was calculated and normalized to the peak height of 2-isopropylmalic acid as an internal standard. Names were assigned to each metabolite peak based on the method described in a previous report{Tsugawa, 2011 #28}.

**4. Evaluation items**

1) Patient characteristics: gender, age at time of registration, date of birth, and patient identification number.

2) General findings: Performance status, height, weight and vital signs.

3) Tumor findings: pathological diagnosis, and clinical stage (TNM classification).

4) Hematology findings: CBC, TP, Alb, T-Bil, AST, ALT, LDH, Amy, BUN, Cr, T-Chol, LDL, HDL, TG, CRP, Glu, HbA1c and Tumor markers including CEA, CA19-9, and DUPAN-2.

5) Concomitant medications.

**5. Endpoint and Statistical considerations**

**5.1. Endpoint**

Primary endpoint：identification of cachexia-related metabolites by metabolomics.

Secondary endpoints：intra-day variation in the metabolites involved in cachexia; changes in the level of serological markers involved in cachexia, such as inflammatory cytokines (e.g. IL-6, TNFα) and leptin, in the presence or absence of cachexia; and changes in QOL survey results in the presence or absence of cachexia.

**5.2. Rationale for sample size**

The present study featured 10 subjects per group, as although this study was exploratory in nature and therefore involved no statistical rationale for sample size calculation, preceding studies on metabolomics have indicated significant results with a sample size of approximately 10 subjects per group. However, if a more useful analysis method found is found that may be implemented in the study, the analysis methods may be altered and the necessary sample size recalculated.

**5.3. Statistical analysis**

Data are expressed as the mean ± standard error. Levels of serological markers and metabolites between the cohorts with and without cachexia were compared using the Mann-Whitney U test or Wilcoxon Signed-Rank test. Kruskal-Wallis multiple comparison (z-test) with bonferroni correction was then used to compare values for each metabolite across all 4 time points tested to determine if there are intra-day variances in the metabolite. Survival time was estimated using the Kaplan-Meier method.

**6. Study institution**

Division of Medical Oncology/Hematology, Kobe University Graduate School of Medicine

Division of Metabolomics Research, Kobe University Graduate School of Medicine

**7. Study period**

From October 2009 to September 2012

**8. Trial registration**

Trial registration ID：UMIN000002384

Date of registration: 01 October 2009

User name: Yutaka Fujiwara

**9. Ensuring study safety**

**9.1. Basic precautions to ensure safety of subjects**

During the study, the study director (attendant physician) must appropriately observe the subjects when necessary, and note their safety. Upon signs of adverse events, appropriate action should be taken as required to ensure the safety of subjects, and the study director must strive to find the cause.

**10. Ethical matters**

**10.1. Protection of subjects**

In carrying out the study, maximum protection of subjects’ human rights, welfare, and safety will be provided in compliance with the ethical principles of the "Declaration of Helsinki" and "Ethical Guidelines for Clinical Research" (Ministry of Health, Labor and Welfare, July 31, 2008). When adverse events, study results, or other relevant data of the study are disclosed, subjects’ personal information will be kept confidential and due consideration for protection of human rights will be given.

**10.2. Informed consent**

**10.2.1. Explanation to subjects**

The investigator is responsible for ensuring that the patient understands the potential risks and benefits of participating in the study, including answering any questions the patient may have throughout the study and sharing in a timely manner any new information that may be relevant to the patient’s willingness to continue his or her participation in the study in a timely manner.

The informed consent form will be used to explain the potential risks and benefits of study participation to the patient in simple terms before the patient is entered into the study and to document that the patient is satisfied with his or her understanding of the potential risks and benefits of participating in the study and desires to participate in the study.

**10.2.2. Informed consent document and form**

Prior to obtaining consent, the following matters will be explained with reference to the document for informed consent form:

- Study overview
- Study purpose
- Study methods
- The mutation analysis of related genes
- Study period
- Planned number of subjects
- Policy for sample handling during and after the study period
- Expected clinical benefits (effects) and disadvantages (side effects)
- Implementing institution of the study
- Therapies that can be received in occurrence of health hazards
- Arbitrary of participation in the study
- Freedom of consent withdrawal from study
- Disclosure of new critical information
- Conditions where participation in the study is discontinued
- Personal information protection poricy
- Publication of study results
- Ownership of intellectual property rights
- Allocation of expenses
- Presence or absence of reward
- Disclosure of study protocol and other materials
- Compliance rules
- Names, job titles, and contact information of the investigators
- Inquiry counter

**10.2.3. Patient consent**

After explanation of the study, patients should be confirmed their full understanding of the study. Thereafter, all patients provided written informed consent after the physician and patient record the date and sign the informed consent form, the physician will hand over a copy of the consent form to the subject, and store the originals in the patient’s medical records.

**10.3. Disclosure of study results**

If this study has a serious effect on the treatment of subjects or on medical profits, we will again explain about study results.

**10.4. Disclosure of study protocol**

If a subject or his/her relatives wishes the study protocol to be disclosed, it will be disclosed as necessary.

**10.5. Storage and management of collected samples**

Collected samples will be stored immediately in a deep freezer at research facilities and managed appropriately.

**10.6. Management of personal identification information**

Samples will be managed and anonymized at the time of registration by the personal data manager. The personal data and case-coding correspondence table will be stored to ensure that only the personal data manager can access them.

**10.7. Approval by Institutional Review Board**

This study approval was obtained from the Institutional Review Board of Kobe University Hospital.

**11. Publication of study results**

Study results will be published or presented in academic conferences by study investigators.

**12. Research funding**

This study was supported by a grant for Research on Applying Health Technology from the Ministry of Health, Labour, and Welfare of Japan.

**13. Ownership of intellectual property rights**

　Ownership of intellectual property rights attribute to Kobe University or investigators.

**14. Investigators**

**Principle investigator:** Prof. Hironobu Minami

Division of Medical Oncology/Hematology, Kobe University Graduate School of Medicine

**Sub-investigators:**

Toru Mukohara, Yutaka Fujiwara, Naomi Kiyota, Masanori Toyoda, Naoko Chayahara, Yoshinori Imamura, Takashi Kobayashi, Shin Nishiumi, Takeshi, Azuma, Masaru Yoshida

**Personal data manager:** Eiichi Maeda

Division of Medical Informatics and Bioinformatics, Kobe University Graduate School of Medicine

**Research secretariat:** Yutaka Fujiwara

Division of Medical Oncology/Hematology, Kobe University Graduate School of Medicine

**15.　References**

1 Uomo G, Gallucci F, Rabitti PG: Anorexia-cachexia syndrome in pancreatic cancer: Recent development in research and management. JOP 2006;7:157-162.

2 Tisdale MJ: Mechanisms of cancer cachexia. Physiol Rev 2009;89:381-410.

3 Hastings M, O'Neill JS, Maywood ES: Circadian clocks: Regulators of endocrine and metabolic rhythms. J Endocrinol 2007;195:187-198.

4 Liu AC, Lewis WG, Kay SA: Mammalian circadian signaling networks and therapeutic targets. Nat Chem Biol 2007;3:630-639.
