# Supplementary material for: Metabolomics Evaluation of Serum Markers for Cachexia and Their Intra-Day Variation in Patients with Advanced Pancreatic Cancer
Source: PLoS One. 2014 Nov 20;9(11):e113259. doi: 10.1371/journal.pone.0113259 (PMC4239056; doi:10.1371/journal.pone.0113259)
Supplement: Statement from Their Ethics Committee or Institutional Review Board S1 — (PDF) [file pone.0113259.s005.pdf]

別紙様式第５

＊受付番号 第 ９４７号

審査結果通知書

平成２１年１０月１５日

申請者
南 博信 殿

神戸大学大学院医学研究科長

高 井 義 美

研 究 課 題　膵臓がん患者における悪液質とそれに関わる代謝産物の日内変動に関するメタボローム解析

研究実施責任者
所 属 職 氏 名　腫瘍・血液内科学　特命教授　南 博信

上記の実施計画について，審査の結果，下記のとおり判定したので通知します。

記

判 定	承 認
理由等	
